# Five Fractions versus Seven Fractions SBRT for Intermediate- and High-Risk Prostate Cancer: A Propensity Score Matched Pair Analysis

**DOI:** 10.3390/cancers15245815

**Published:** 2023-12-12

**Authors:** Christina Schröder, Lucas Mose, Etienne Mathier, Daniel Rudolf Zwahlen, Daniel Matthias Aebersold, Robert Förster, Mohamed Shelan

**Affiliations:** 1 Department of Radiation Oncology, Cantonal Hospital Winterthur, 8400 Winterthur, Switzerland; 2 Department of Radiation Oncology, Inselspital/Bern University Hospital, University of Bern, CH-3010 Bern, Switzerland; lucas.mose@insel.ch (L.M.); mohamed.shelan@insel.ch (M.S.)

**Keywords:** prostate cancer, stereotactic body radiotherapy, propensity score, disease-free survival

## Abstract

**Simple Summary:**

Stereotactic body radiotherapy (SBRT) is a contemporary treatment option for prostate cancer with excellent efficacy and low toxicity. Traditionally, this treatment option was used for patients with low- to intermediate-risk prostate cancer but there is emerging data for the high-risk setting. However, the optimal fractionation schedule for prostate SBRT remains an interesting research question. We therefore compared two cohorts that received radiotherapy with different treatment schedules regarding efficacy and toxicity. These patients included in these cohort were specifically matched, so that they would be similar.

**Abstract:**

Purpose: To compare two stereotactic body radiotherapy (SBRT) regimens in patients with intermediate- or high-risk prostate cancer with regards toxicity and efficacy. Methods/Material: We retrospectively collected data from 198 patients treated with SBRT for prostate cancer at two different institutions. Patients received either 35–36.25 Gy in five fractions (group A) using Cyberknife robotic platform or 42.7 Gy in seven fractions (group B) using a C-arm LINAC (image-guided). Propensity score matching was done (2:1 nearest neighbor matching without replacement), resulting in 120 patients (80 patients for group A, 40 patients for group B). Toxicity, PSA nadir, biochemical failure and disease-free survival (DFS) were analyzed. Results: Median follow up of all patients was 13 months (range 1–91 months). Overall, 23.3% of patients had ≥G2 acute GU toxicity (21.1% group A versus 30% group B (*p* = 0.222)) and 6.6% of patients ≥G2 GI toxicity (2.5% versus 15% (*p* = 0.010)). There was one acute G3 GU toxicity in arm A and one acute G4 rectal bleeding in group B (anticoagulated patient). Regarding late toxicity, 14.1% of patients had ≥G2 late GU toxicity (17.4% versus 6.6% (*p* = 0.159)) and 5.0% of patients had ≥G2 late GI toxicity (1.4% versus 13.3% (*p* = 0.013)). There was one G3 late GU toxicity in arm B and two G3 late GI toxicities, one in each arm. Relative median PSA reduction was 92.4% (−53.9–99.9%) from baseline PSA (93.7% (−53.9–99.9%) in group A versus 87.7% (39.8–99.9%) in group B (*p* = 0.043). In total, 4.2% of patients had biochemical relapse, 5.0% in group A and 2.5% in group B (*p* = 0.518). One-year DFS in the overall cohort was 97.3%, 98.8% in group A and 94.3% in group B (*p* = 0.318). Conclusion: Both SBRT regimens have acceptable acute and late toxicity and good efficacy. There are significantly more GI toxicities in the seven-fraction regimen. Longer follow-up is warranted for better comparison of long-term efficacy.

## 1. Introduction

Prostate cancer (PC) is the second most common cancer in men with an estimated incidence of 1.4 million new patients worldwide in 2020 [1]. Active treatment options for localized disease include radical prostatectomy (RPE) and radiotherapy (RT) with or without androgen deprivation therapy (ADT) [2]. Both approaches offer comparable therapeutic efficacy by reducing metastatic incidence by 50% compared to active surveillance while achieving excellent local control and quality of life [2]. Nevertheless, they exhibit specific advantages and disadvantages due to the different side-effect profiles and logistic load.

A significant drawback of conventional external beam RT is the large number of treatment sessions required for a complete RT course, typically extending up to 2 months. To mitigate this time commitment, shortened treatment schedules with slightly higher single dose per fraction have been explored. Results from randomized studies have demonstrated that these moderately hypofractionated regimens, with a dose ranging from 2.5 to 3 Gy per fraction, are not inferior to conventional standard schedules in terms of biochemical control and tolerability [3,4,5].

Stereotactic Body Radiotherapy (SBRT) represents a more contemporary treatment option, enabled by precise positioning technologies that allow for higher single-dose delivery per fraction. According to guidelines from ASTRO, ASCO, and AUA, ultra-hypofractionated radiation therapy is defined as a dosage of 5.0 Gy per day or higher [6]. This approach can be particularly effective due to the estimated low α/β value of prostate cancer of around 1.5 Gy [7,8]. Therefore, SBRT has been used as a treatment option in patients with low or intermediate risk for a long time and there are published data with a reasonable follow-up that show excellent biochemical control with low high-grade toxicity rates [9,10,11,12,13,14,15,16,17]. In addition, data on SBRT in high-risk patients are emerging with several publications showing encouraging results [14,18,19,20,21,22,23].

However, the optimal fractionation schedule and total treatment dose to be delivered in the frame of SBRT remains unknown and therefore an interesting research question. In this retrospective propensity score matched pair analysis, we aim to compare two different fractionation regimens used in two different departments, focusing on the toxicity outcome and PSA response after treatment.

## 2. Methods and Materials

### 2.1. Stereotactic Radiotherapy

Patients received either 35–36.25 Gy in 5 fractions (group A) on Cyberknife robotic platform or 42.7 Gy in 7 fractions (group B) using image-guided RT on a C-arm Linac.

In group A, RT was delivered by Cyberknife (Accuray, Inc., Sunnyvale, CA, USA). Four gold fiducial makers were placed in the prostate for real-time motion tracking 2 weeks before treatment. The prescription dose was 35–36.25 Gy in five fractions delivered every other day. Treatment planning was performed using CT scan fused to MRI images. The clinical target volume (CTV) included prostate and seminal vesicles base (1 cm) depending on the risks. A margin of 5 mm in all directions and 3 mm posteriorly was added to the CTV to generate the planning target volume (PTV). The prescribed dose was normalized to the 75–85% isodose line (IDL) for patients with prostate cancer.

In group B, patients received a prescription according to Widmark et al. (42.7 Gy in 7 fractions) [22]. RT was delivered with image-guided volumetric modulated arc therapy (VMAT) using daily cone-beam CT (CBCT). Dose prescription was homogeneous. Patients were given detailed instructions for bladder filling and rectal balloons were used for additional immobilization of the prostate region. The CTV was defined as prostate only with a 7 mm margin to derive the PTV. Depending on risk factors, the base of the seminal vesicles were included. In total, 97.5% of patients had an additional MRI for treatment planning. 60% of patients received an additional PSMA PET CT for staging, another 10% received staging using bone scintigraphy and CT thorax/abdomen. Treatment was delivered on alternating days, three days per week.

In both groups, ADT was given at the discretion of the treating physician.

### 2.2. Patient Characteristic

Median age of patients was 71.8 years (range 52–85 years). Most patients (78.3%) had intermediate-risk prostate cancer with Gleason 7 being the most prominent Gleason score (78.3%). Median prostate volume was 45.1 cc (range 20–102 cc). Prostate volume and the prevalence of hypertension were the only patient-related characteristics that differed between the groups. Further information can be found in Table 1.

### 2.3. Treatment Characteristics

Overall, 25 patients (20.8%) received ADT; 20 patients in group A (25% of patients in group A) and 5 patients (12.5%) in group B. 19 patients received short term ADT (up to 6 months) while 6 patients received long-term ADT (≥18 months). Further treatment characteristics can be found in Table 2.

### 2.4. Statistical Analysis

This analysis was approved by the local ethics committees (Canton of Zürich BASEC 2020-02112, Canton of Bern 2022-00120).

Propensity matching analysis was carried out with R (version 4.2.2.). Propensity score was estimated with logistic regression using the following variables: IPSS, risk group and prostate volume. For this, 2:1 nearest neighbor matching without replacement was used. This resulted in 40 patients treated in seven fractions and 80 patients treated in 5 fractions from an original dataset of 198 patients.

Toxicity was scored according to the Common Terminology Criteria for Adverse Events (CTCAE) version 5. Acute toxicity was defined as toxicity within the first 3 months after the start of treatment, late toxicity was defined as toxicity occurring more than 3 months after start of RT. Time to nadir was defined as the time from the start of treatment until nadir, disease free survival (DFS) was defined from the start of treatment until recurrence or death.

Descriptive statistics were done as well as Chi Square test and Mann–Whitney U Test for group comparison. For correlation, Spearman correlation was used and DFS was calculated using the Kaplan–Meier method.

For the statistical analyses IBM SPSS, version 29 (Statistical Package for Social Sciences, International Business Machines Corp., Armonk, NY, USA) was used.

## 3. Results

### 3.1. Acute and Late Toxicity

Overall, 23.3% of patients had ≥grade 2 (G2) acute genitourinary (GU) toxicity and 6.6% of patients had ≥G2 acute gastrointestinal (GI) toxicity. When looking at the groups separately, 21.1% of patients in group A had ≥G2 GU toxicity versus 30% in group B (*p* = 0.222). For ≥G2 GI toxicity those numbers were 2.5% for group A and 15% for group B (*p* = 0.010). There were two ≥G3 acute toxicities, one acute G3 GU toxicity in arm A and an acute G4 rectal bleeding in group B. This patient was anticoagulated and simultaneously had an additional source of bleeding in the stomach.

Regarding late toxicity, 14.1% of patients had ≥G2 late GU toxicity and 5.0% of patients had ≥G2 late GI toxicity in the overall cohort. For the two groups, these values were 17.4% for group A versus 6.6% for group B for late GU toxicity (*p* = 0.159) and 1.4% for group A versus 13.3% for group B for late GI toxicity (*p* = 0.013). There was one G3 late GU toxicity in arm B and two G3 late GI toxicities, one in each arm.

### 3.2. Nadir and Disease Free Survival

Median follow-up of all patients was 13 months (range: 1–91 months). Median PSA nadir in the overall cohort was 0.6 ng/mL (range <0.01–5.88 ng/mL), 0.5 ng/mL in group A and 0.9 ng/mL in group B (*p* = 0.018). When looking at patients with a follow-up of 1 year or more, median PSA nadir was 0.50 ng/mL (range <0.01–4.7 ng/mL), 0.50 ng/mL in group A and 0.56 ng/mL in group B (*p* = 0.247).

The relative median PSA reduction was 92.4% (range −53.9–99.9%) from baseline PSA. For group A the median reduction was 93.7% (range −53.9–99.9%) versus 87.7% (range 39.8–99.9%) in group B (*p* = 0.043).

**Figure 1:**
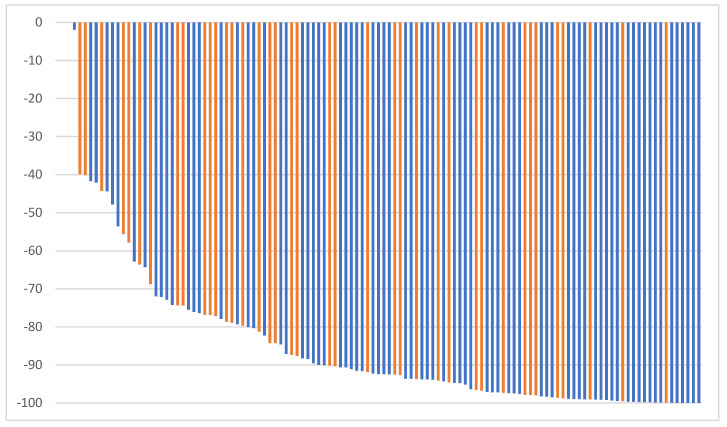
Maximum PSA reduction from baseline (group A: blue, group B: orange).

The median time to nadir was 9 months (range 1–88 months) for all patients (10.0 months (range 1–88 months) group A, 8 months (range 1–34 months) group B). Figure 1 shows a waterfall plot of the PSA reduction from baseline excluding one patient with immediate relapse after SBRT.

**Figure 2:**
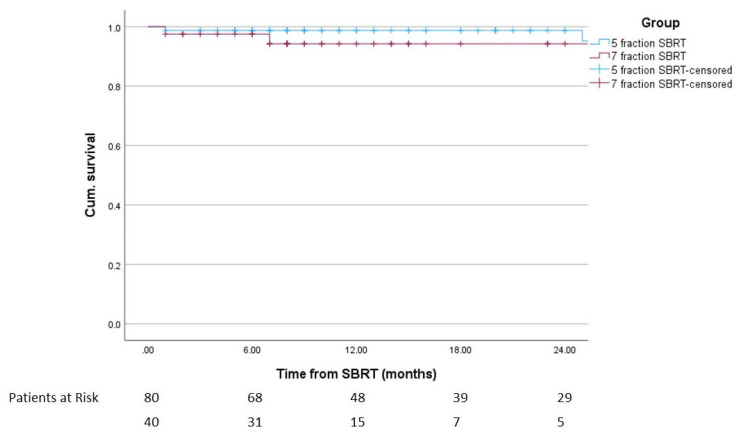
Disease-free survival after the start of SBRT including patients at risk (*p* = 0.236).

Overall, 4.2% of patients had biochemical relapse, 5.0% in group A and 2.5% in group B (*p* = 0.518). All patients with biochemical relapse also had clinical relapse. One-year DFS in the overall cohort was 97.3%, 98.8% in group A and 94.3% in group B (*p* = 0.318). Of the patients with clinical relapse, one patient in group A had local recurrence, two patients in group A had local and regional recurrence and the remaining two patients (one in each group) had distant recurrence only. Figure 2 shows the disease free survival.

Overall, four patients died (one tumor related, three non-tumor related), two in each arm with the tumor-related death in group B. One-year OS was 97.3% in the overall cohort, with 98.8% and 94.3% in the groups, respectively (*p* = 0.206).

## 4. Discussion

The current analysis is a matched-pairs analysis comparing the five-fraction and the seven-fraction SBRT regimens for localized prostate cancer. To the best of our knowledge, such an analysis has not been reported, yet.

Most studies on prostate SBRT included patients with low or intermediate risk. There is no level 1 evidence for patients with high-risk prostate cancer but there is an increasing level of prospective data, so that SBRT may be offered to selected high-risk patients [20]. The optimal fractionation scheme for prostate SBRT remains a topic of research. Traditionally, most studies used a five-fraction regimen. Compared to the commonly used 5 × 7.25 Gy fractionation scheme, Widmark et al. used a slightly higher treatment dose in the Hypo-RT-PC trial, which included patients with intermediate- and high-risk prostate cancer [22]. More recently, there have been other approaches including, e.g., one-fraction regimens or using a simultaneous integrated boost to the dominant intraprostatic lesion (DIL) [14,23,24,25,26,27]. Boosting the DIL seems a promising approach as most recurrences appear in the PI RADS 4-5 DIL which seems especially appropriate in the high-risk setting [28]. Nevertheless, published data on both fractionation schemes discussed in this publication show good efficacy and low toxicity [16,22].

In this cohort, overall acute ≥G2 GU toxicity was seen in 23.3% of patients and up to 30% of patients receiving the seven-fraction regimen. This remains in line with the range of the published literature [10,14,19,22,29,30,31]. Notably, there is little toxicity data for the seven-fraction regimen with the original trial by Widmark et al. showing 28% acute ≥G2 GU toxicity [22]. Late ≥G2 GU toxicity was seen in 14.1% of patients with up to 17.4% of patients in group A. These values are within the published range as well [10,11,19,22,29,30,31]. There was no statistically significant difference between both treatment groups, although the dosimetric data for bladder and urethra are significantly higher in the seven-fraction regimen (see Table 2).

Acute ≥G2 GI toxicity was 6.6% in the overall cohort with a significantly higher in group B with 15%. This remained similar for late ≥G2 toxicity. Notably, the 15% GI toxicity were still within the published range by Widmark et al. reporting around 10% GI toxicity and the PACE-B trial reporting up to 15.6% acute ≥G2 GI toxicity [16,22]. Most other SBRT studies report late GI toxicity of up to 10% [14,29,31,32]. The significant differences in ≥G2 toxicity are likely explained by the higher median rectal dose (see Table 2). The patients in group B were contoured according to Widmark et al. with a 7 mm margin to derive the PTV, while patients in group A had a 3 mm margin towards the rectum. Therefore, the higher dose to the rectum was to be expected. Additionally, more patients in group B suffered from T3, especially T3b disease resulting in significantly larger CTVs than in group A. However, with the rectal planning objectives from the original trial by Widmark et al., the overall risk for late rectal toxicity can still be considered reasonably low [22,33] and with strict image guidance the dorsal margin could be reduced.

Another factor to consider is the different total treatment dose of the two regimens, especially when considering the EQD2 dose. With an alpha/beta value of three as commonly used for organs at risk (OAR), this results in 74.31 Gy_3_ for group A and 77.71 Gy_3_ for group B; meaning a higher dose for OAR in group B. This might be especially relevant for OAR that are within the PTV like the urethra or parts of the bladder. However, there was no statistically significant difference in GU toxicity in this analysis.

Patients were treated on different treatment machines with different IGRT modalities. Patients in group A were treated at a CK using fiducials while patients in group B were treated on a C-arm LINAC using rectal balloons and daily CBCT. For Cyberknife patients, there is a tendency to use smaller PTV margins like in our cohort. Ito et al. conducted a propensity score analysis comparing 3-year GU toxicity in prostate cancer patients treated with SBRT either using a Cyberknife or a Linac-based VMAT with PTV margins of 3 mm (1 mm posterior) for Cyberknife or 6 mm (3 mm posterior) for VMAT. They found a tendency to lower acute G2 GU toxicity for patients treated with Cyberknife but no difference in late G2 GU or GI toxicity [34]. In the Pace B trial, patients that were treated with a CK had significantly lower worst GU and GI toxicity at 24 months. However, this difference was only significant if toxicity was scored according to CTCAE but not when it was scored according to RTOG [16]. Additionally, more patients that were treated with a CK had low-risk disease, resulting in smaller targets due to the exclusion of the base of the seminal vesicles according to the protocol and a lower rate of alpha-blockers at baseline [16]. The overall value of fiducials compared to daily CBCT with matching of the prostate region is somewhat unclear. Although there might be additional benefit, the overall variability when using CBCT seems acceptably low [35,36,37,38].

The median nadir for patients with 1-year follow-up was 0.6 ng/mL. This value is within published range for this time point and is expected to reduce further with increasing follow-up [39,40,41,42,43]. Median time to nadir after SBRT was between 30 and 45 months in other SBRT cohorts, although the maximum PSA decline seems to be within the first year after SBRT [39,40,41,42,43]. Some studies suggest that the PSA nadir and time to nadir may have a prognostic impact [40,41,42].

Biochemical recurrence in the overall cohort was 4.2% and 1-year DFS was 97.3%. These values are overall well within the range of published data for cohorts including intermediate and high-risk patients [14,19,22,23,32]. Isolated local recurrence only occurred in one patient. The other patients had either locoregional, regional of metastatic recurrence with no difference between the groups. Notably, ADT was given in 20% of patients with only 5% receiving long term ADT.

There are several key limitations to this study which mostly lie in the retrospective nature and the limited follow up especially in group B. However, the latter can be explained by the publication date of the Hypo-RT-PC study in 2019 [22] and the consecutive publication of the quality of life data in 2021 [44], after which the regimen was adopted into clinical routine. The short follow-up might somewhat bias the results, especially in patients receiving ADT. Additionally, the application method differed between both groups as well as the definition and size and margins of the target volumes (including a higher amount of T3b patients in group B.

Still, to the best of our knowledge this is the first matched-pairs analysis comparing the five-fraction and seven-fraction SBRT regimen for localized prostate cancer.

## 5. Conclusions

In conclusion, SBRT regimens using five or seven fractions appear to have acceptable acute and late toxicity as well as good efficacy. There are significantly more ≥GI toxicities in the seven-fraction regimen, likely the result of the higher overall treatment dose, the overall larger CTVs and the larger PTV margins. Longer follow-up is warranted for better comparison of long-term efficacy.

## Figures and Tables

**Table 1:** Patient characteristics.

	Group A (5 Fractions)	Group B (7 Fractions)	All Patients
	N (%)	N (%)	N (%)
IPSS before SBRT			
<7	53 (66.2)	26 (65.0)	79 (65.8)
≥7	27 (33.8)	14 (35.0)	41 (34.2)
TUR-P			
No	74 (92.5)	37 (92.5)	111 (92.5)
Yes	6 (7.5)	3 (7.5)	9 (7.5)
Hypertension			
No	51 (63.8)	18 (45.0)	69 (57.5)
Yes	29 (36.2)	22 (55.0)	51 (42.5)
Anticoagulation			
No	56 (70.0)	28 (70.0)	84 (70.0)
Yes	24 (30.0)	12 (30.0)	36 (30.0)
Diabetes			
No	66 (82.5)	34 (85.0)	100 (83.3)
Yes	14 (17.5)	6 (15.0)	20 (16.7)
NCCN-risk group			
Low risk	5 (6.2)	5 (12.5)	10 (8.3)
Favorable intermediate risk	28 (35.0)	9 (22.5)	37 (30.8)
Unfavorable intermediate risk	32 (40.0)	16 (40.0)	48 (40.0)
High risk	15 (18.8)	10 (25.0)	25 (20.8)
T-Stage *			
T1a/T1b/T1c	30 (37.5)	3 (7.5)	33 (27.5)
T2a/T2b/T2c	44 (55.0)	28 (70.0)	72 (60.0)
T3a/T3b	6 (7.5)	9 (22.5)	15 (12.5)
Gleason Score			
6	9 (11.2)	11 (27.5)	20 (16.7)
7a	46 (57.5)	17 (42.5)	63 (52.5)
7b	21 (26.3)	10 (25.0)	31 (25.8)
≥8	4 (5.0)	2 (5.0)	6 (5.0)
Total	80 (100)	40 (100)	120 (100)
	Median (range)	Median (range)	
Age at SBRT (years)	72 (54–85)	72 (55–88)	
Prostate Volume (cc) *	45.4 (20.0–102.0)	52.8 (22.0–99.8)	

* *p* < 0.05.

**Table 2:** Treatment characteristics.

	Group A (5 Fractions)	Group B (7 Fractions)	All Patients
	N (%)	N (%)	N (%)
Duration ADT			
None	60 (75.0)	35 (87.5)	95 (79.2)
3 months	2 (2.5)	0 (0.0)	2 (1.7)
6 months	13 (16.3)	4 (10.0)	17 (14.2)
≥18 months	5 (6.3)	1 (2.5)	6 (5.0)
Fractionation			
5 × 7 Gy (80% IDL)	46 (57.5)	0 (0.0)	46 (38.3)
5 × 7.25 Gy (77.8–80% IDL)	34 (42.5)	0 (0.0)	34 (28.3)
7 × 6.1 Gy	0 (0.0)	40 (100)	40 (33.3)
	Median (range)	Median (range)	
PTV volume (cc) *	95.1 (48.0–140.6)	137.0 (84.9–233.9)	
CTV volume (cc) *	51 (22–102 cc)	57.5 (33.2–107.3)	
Rectum D1cc (Gy) *	34.7 (27.3–38.6)	43.9 (43.6–44.5)	
Bladder D10cc (Gy) *	32.6 (18.9–36.3)	42.1 (32.5–42.9)	
Urethra Dmax (Gy) *	41.0 (35.9–43.4	44.1 (43.0–45.0)	
Bowel Dmax (Gy) *	13.2 (6.0–34.4)	30.0 (16.0–43.0)	

* *p* < 0.05.

## Data Availability

The data are not publicly available due to ethical reasons.

## References

[1] Sung H., Ferlay J., Siegel R.L., Laversanne M., Soerjomataram I., Jemal A., Bray F. (2021). Global Cancer Statistics 2020: GLOBOCAN Estimates of Incidence and Mortality Worldwide for 36 Cancers in 185 Countries. CA Cancer J. Clin..

[2] Hamdy F.C., Donovan J.L., Lane J.A., Metcalfe C., Davis M., Turner E.L., Martin R.M., Young G.J., Walsh E.I., Bryant R.J. (2023). Fifteen-Year Outcomes after Monitoring, Surgery, or Radiotherapy for Prostate Cancer. N. Engl. J. Med..

[3] Lee W.R., Dignam J.J., Amin M.B., Bruner D.W., Low D., Swanson G.P., Shah A.B., D’Souza D.P., Michalski J.M., Dayes I.S. (2016). Randomized Phase III Noninferiority Study Comparing Two Radiotherapy Fractionation Schedules in Patients with Low-Risk Prostate Cancer. J. Clin. Oncol..

[4] Dearnaley D., Syndikus I., Mossop H., Khoo V., Birtle A., Bloomfield D., Graham J., Kirkbride P., Logue J., Malik Z. (2016). Conventional versus hypofractionated high-dose intensity-modulated radiotherapy for prostate cancer: 5-year outcomes of the randomised, non-inferiority, phase 3 CHHiP trial. Lancet Oncol..

[5] Catton C.N., Lukka H., Gu C.S., Martin J.M., Supiot S., Chung P.W.M., Bauman G.S., Bahary J.P., Ahmed S., Cheung P. (2017). Randomized Trial of a Hypofractionated Radiation Regimen for the Treatment of Localized Prostate Cancer. J. Clin. Oncol..

[6] Morgan S.C., Hoffman K., Loblaw D.A., Buyyounouski M.K., Patton C., Barocas D., Bentzen S., Chang M., Efstathiou J., Greany P. (2019). Hypofractionated Radiation Therapy for Localized Prostate Cancer: Executive Summary of an ASTRO, ASCO and AUA Evidence-Based Guideline. J. Urol..

[7] Brenner D.J., Martinez A.A., Edmundson G.K., Mitchell C., Thames H.D., Armour E.P. (2002). Direct evidence that prostate tumors show high sensitivity to fractionation (low alpha/beta ratio), similar to late-responding normal tissue. Int. J. Radiat. Oncol. Biol. Phys..

[8] Miralbell R., Roberts S.A., Zubizarreta E., Hendry J.H. (2012). Dose-fractionation sensitivity of prostate cancer deduced from radiotherapy outcomes of 5,969 patients in seven international institutional datasets: Alpha/beta = 1.4 (0.9–2.2) Gy. Int. J. Radiat. Oncol. Biol. Phys..

[9] Boike T.P., Lotan Y., Cho L.C., Brindle J., DeRose P., Xie X.J., Yan J., Foster R., Pistenmaa D., Perkins A. (2011). Phase I dose-escalation study of stereotactic body radiation therapy for low- and intermediate-risk prostate cancer. J. Clin. Oncol..

[10] Brand D.H., Tree A.C., Ostler P., van der Voet H., Loblaw A., Chu W., Ford D., Tolan S., Jain S., Martin A. (2019). Intensity-modulated fractionated radiotherapy versus stereotactic body radiotherapy for prostate cancer (PACE-B): Acute toxicity findings from an international, randomised, open-label, phase 3, non-inferiority trial. Lancet Oncol..

[11] Chen L.N., Suy S., Uhm S., Oermann E.K., Ju A.W., Chen V., Hanscom H.N., Laing S., Kim J.S., Lei S. (2013). Stereotactic body radiation therapy (SBRT) for clinically localized prostate cancer: The Georgetown University experience. Radiat. Oncol..

[12] Jackson W.C., Silva J., Hartman H.E., Dess R.T., Kishan A.U., Beeler W.H., Gharzai L.A., Jaworski E.M., Mehra R., Hearn J.W.D. (2019). Stereotactic Body Radiation Therapy for Localized Prostate Cancer: A Systematic Review and Meta-Analysis of Over 6000 Patients Treated On Prospective Studies. Int. J. Radiat. Oncol. Biol. Phys..

[13] King C.R., Brooks J.D., Gill H., Presti J.C. (2012). Long-term outcomes from a prospective trial of stereotactic body radiotherapy for low-risk prostate cancer. Int. J. Radiat. Oncol. Biol. Phys..

[14] Kotecha R., Djemil T., Tendulkar R.D., Reddy C.A., Thousand R.A., Vassil A., Stovsky M., Berglund R.K., Klein E.A., Stephans K.L. (2016). Dose-Escalated Stereotactic Body Radiation Therapy for Patients With Intermediate- and High-Risk Prostate Cancer: Initial Dosimetry Analysis and Patient Outcomes. Int. J. Radiat. Oncol. Biol. Phys..

[15] Meier R.M., Bloch D.A., Cotrutz C., Beckman A.C., Henning G.T., Woodhouse S.A., Williamson S.K., Mohideen N., Dombrowski J.J., Hong R.L. (2018). Multicenter Trial of Stereotactic Body Radiation Therapy for Low- and Intermediate-Risk Prostate Cancer: Survival and Toxicity Endpoints. Int. J. Radiat. Oncol. Biol. Phys..

[16] Tree A.C., Ostler P., van der Voet H., Chu W., Loblaw A., Ford D., Tolan S., Jain S., Martin A., Staffurth J. (2022). Intensity-modulated radiotherapy versus stereotactic body radiotherapy for prostate cancer (PACE-B): 2-year toxicity results from an open-label, randomised, phase 3, non-inferiority trial. Lancet Oncol..

[17] Zilli T., Franzese C., Bottero M., Giaj-Levra N., Forster R., Zwahlen D., Koutsouvelis N., Bertaut A., Blanc J., Roberto D’agostino G. (2019). Single fraction urethra-sparing prostate cancer SBRT: Phase I results of the ONE SHOT trial. Radiother. Oncol..

[18] Bauman G., Ferguson M., Lock M., Chen J., Ahmad B., Venkatesan V.M., Sexton T., D’Souza D., Loblaw A., Warner A. (2015). A Phase 1/2 Trial of Brief Androgen Suppression and Stereotactic Radiation Therapy (FASTR) for High-Risk Prostate Cancer. Int. J. Radiat. Oncol. Biol. Phys..

[19] Callan L., Bauman G., Chen J., Lock M., Sexton T., D’Souza D., Rodrigues G. (2019). A Phase I/II Trial of Fairly Brief Androgen Suppression and Stereotactic Radiation Therapy for High-Risk Prostate Cancer (FASTR-2): Preliminary Results and Toxicity Analysis. Adv. Radiat. Oncol..

[20] Foerster R., Zwahlen D.R., Buchali A., Tang H., Schroeder C., Windisch P., Vu E., Akbaba S., Bostel T., Sprave T. (2021). Stereotactic Body Radiotherapy for High-Risk Prostate Cancer: A Systematic Review. Cancers.

[21] Murthy V., Gupta M., Mulye G., Maulik S., Munshi M., Krishnatry R., Phurailatpam R., Mhatre R., Prakash G., Bakshi G. (2018). Early Results of Extreme Hypofractionation Using Stereotactic Body Radiation Therapy for High-risk, Very High-risk and Node-positive Prostate Cancer. Clin. Oncol. (R. Coll. Radiol.).

[22] Widmark A., Gunnlaugsson A., Beckman L., Thellenberg-Karlsson C., Hoyer M., Lagerlund M., Kindblom J., Ginman C., Johansson B., Bjornlinger K. (2019). Ultra-hypofractionated versus conventionally fractionated radiotherapy for prostate cancer: 5-year outcomes of the HYPO-RT-PC randomised, non-inferiority, phase 3 trial. Lancet.

[23] Zilli T., Jorcano S., Bral S., Symon Z., Rubio C., Bruynzeel A.M.E., Ibrahimov R., Minn H., Oliveira A., Bertaut A. (2023). Every-Other-Day Versus Once-a-Week Urethra-Sparing Prostate Stereotactic Body Radiation Therapy: 5-Year Results of a Randomized Phase 2 Trial. Int. J. Radiat. Oncol. Biol. Phys..

[24] Draulans C., van der Heide U.A., Haustermans K., Pos F.J., van der Voort van Zyp J., De Boer H., Groen V.H., Monninkhof E.M., Smeenk R.J., Kunze-Busch M. (2020). Primary endpoint analysis of the multicentre phase II hypo-FLAME trial for intermediate and high risk prostate cancer. Radiother. Oncol..

[25] Greco C., Pares O., Pimentel N., Louro V., Santiago I., Vieira S., Stroom J., Mateus D., Soares A., Marques J. (2021). Safety and Efficacy of Virtual Prostatectomy With Single-Dose Radiotherapy in Patients With Intermediate-Risk Prostate Cancer: Results From the PROSINT Phase 2 Randomized Clinical Trial. JAMA Oncol..

[26] Kerkmeijer L.G.W., Groen V.H., Pos F.J., Haustermans K., Monninkhof E.M., Smeenk R.J., Kunze-Busch M., de Boer J.C.J., van der Voort van Zijp J., van Vulpen M. (2021). Focal Boost to the Intraprostatic Tumor in External Beam Radiotherapy for Patients With Localized Prostate Cancer: Results from the FLAME Randomized Phase III Trial. J. Clin. Oncol..

[27] Zilli T., Jorcano S., Bral S., Rubio C., Bruynzeel A.M.E., Oliveira A., Abacioglu U., Minn H., Symon Z., Miralbell R. (2020). Once-a-week or every-other-day urethra-sparing prostate cancer stereotactic body radiotherapy, a randomized phase II trial: 18 months follow-up results. Cancer Med..

[28] Gorovets D., Wibmer A.G., Moore A., Lobaugh S., Zhang Z., Kollmeier M., McBride S., Zelefsky M.J. (2023). Local Failure after Prostate SBRT Predominantly Occurs in the PI-RADS 4 or 5 Dominant Intraprostatic Lesion. Eur. Urol. Oncol..

[29] Fan C.Y., Chao H.L., Huang W.Y., Lin C.S., Chen C.M., Lo C.H. (2015). Stereotactic ablative radiotherapy with CyberKnife in the treatment of locally advanced prostate cancer: Preliminary results. Tumori.

[30] Janowski E., Chen L.N., Kim J.S., Lei S., Suy S., Collins B., Lynch J., Dritschilo A., Collins S. (2014). Stereotactic body radiation therapy (SBRT) for prostate cancer in men with large prostates (>/=50 cm^3^). Radiat. Oncol..

[31] Oliai C., Lanciano R., Sprandio B., Yang J., Lamond J., Arrigo S., Good M., Mooreville M., Garber B., Brady L.W. (2013). Stereotactic body radiation therapy for the primary treatment of localized prostate cancer. J. Radiat. Oncol..

[32] Koskela K., Palmgren J.E., Heikkila J., Virsunen H., Sailas L., Auvinen P., Seppala J., Kataja V. (2017). Hypofractionated stereotactic body radiotherapy for localized prostate cancer—First Nordic clinical experience. Acta Oncol..

[33] Wang K., Mavroidis P., Royce T.J., Falchook A.D., Collins S.P., Sapareto S., Sheets N.C., Fuller D.B., El Naqa I., Yorke E. (2021). Prostate Stereotactic Body Radiation Therapy: An Overview of Toxicity and Dose Response. Int. J. Radiat. Oncol. Biol. Phys..

[34] Ito M., Yoshioka Y., Takase Y., Suzuki J., Takahashi H., Minami Y., Sakuragi A., Oshima Y., Okuda T., Suzuki K. (2023). Stereotactic body radiation therapy for prostate cancer: A study comparing 3-year genitourinary toxicity between CyberKnife and volumetric-modulated arc therapy by propensity score analysis. Radiat. Oncol..

[35] Goff P.H., Harrison L.B., Furhang E., Ng E., Bhatia S., Trichter F., Ennis R.D. (2017). 2D kV orthogonal imaging with fiducial markers is more precise for daily image guided alignments than soft-tissue cone beam computed tomography for prostate radiation therapy. Adv. Radiat. Oncol..

[36] Ye J.C., Qureshi M.M., Clancy P., Dise L.N., Willins J., Hirsch A.E. (2015). Daily patient setup error in prostate image guided radiation therapy with fiducial-based kilovoltage onboard imaging and conebeam computed tomography. Quant. Imaging Med. Surg..

[37] Moseley D.J., White E.A., Wiltshire K.L., Rosewall T., Sharpe M.B., Siewerdsen J.H., Bissonnette J.P., Gospodarowicz M., Warde P., Catton C.N. (2007). Comparison of localization performance with implanted fiducial markers and cone-beam computed tomography for on-line image-guided radiotherapy of the prostate. Int. J. Radiat. Oncol. Biol. Phys..

[38] Yildirim B.A., Onal C., Dolek Y. (2017). Is it essential to use fiducial markers during cone-beam CT-based radiotherapy for prostate cancer patients?. Jpn. J. Radiol..

[39] Anwar M., Weinberg V., Chang A.J., Hsu I.C., Roach M., Gottschalk A. (2014). Hypofractionated SBRT versus conventionally fractionated EBRT for prostate cancer: Comparison of PSA slope and nadir. Radiat. Oncol..

[40] Jiang N.Y., Dang A.T., Yuan Y., Chu F.I., Shabsovich D., King C.R., Collins S.P., Aghdam N., Suy S., Mantz C.A. (2019). Multi-Institutional Analysis of Prostate-Specific Antigen Kinetics After Stereotactic Body Radiation Therapy. Int. J. Radiat. Oncol. Biol. Phys..

[41] Kole T.P., Chen L.N., Obayomi-Davies O., Kim J.S., Lei S., Suy S., Dritschilo A., Collins S.P. (2015). Prostate specific antigen kinetics following robotic stereotactic body radiotherapy for localized prostate cancer. Acta Oncol..

[42] Park Y., Park H.J., Jang W.I., Jeong B.K., Kim H.J., Chang A.R. (2018). Long-term results and PSA kinetics after robotic SBRT for prostate cancer: Multicenter retrospective study in Korea (Korean radiation oncology group study 15-01). Radiat. Oncol..

[43] Rucinska M., Osowiecka K., Kieszkowska Grudny A., Nawrocki S. (2019). SHARP hypofractionated stereotactic radiotherapy for localized prostate cancer: A biochemical response to treatment. J. BUON.

[44] Fransson P., Nilsson P., Gunnlaugsson A., Beckman L., Tavelin B., Norman D., Thellenberg-Karlsson C., Hoyer M., Lagerlund M., Kindblom J. (2021). Ultra-hypofractionated versus conventionally fractionated radiotherapy for prostate cancer (HYPO-RT-PC): Patient-reported quality-of-life outcomes of a randomised, controlled, non-inferiority, phase 3 trial. Lancet Oncol..

